# Innovation in viruses: fitness valley crossing, neutral landscapes, or just duplications?

**DOI:** 10.1093/ve/veae078

**Published:** 2024-09-20

**Authors:** Paul Banse, Santiago F Elena, Guillaume Beslon

**Affiliations:** INSA Lyon, INRIA, CNRS, Universite Claude Bernard Lyon 1, Ecole Centrale de Lyon, Université Lumière Lyon 2, LIRIS, UMR5205, Villeurbanne 69621, France; Instituto de Biología Integrativa de Sistemas (I2SysBio), CSIC-Universitat de València, Catedrático Agustín Escardino 9, Paterna, Valencia 46980, Spain; Santa Fe Institute, 1399 Hyde Park Road, Santa Fe, NM 87501, USA; INSA Lyon, INRIA, CNRS, Universite Claude Bernard Lyon 1, Ecole Centrale de Lyon, Université Lumière Lyon 2, LIRIS, UMR5205, Villeurbanne 69621, France

**Keywords:** chromosomal rearrangements, combinatorics, fitness landscape, innovation, structural variation

## Abstract

Viruses evolve by periods of relative stasis interleaved with sudden, rapid series of mutation fixations, known as evolutionary bursts. These bursts can be triggered by external factors, such as environmental changes, antiviral therapies, or spill-overs from reservoirs into novel host species. However, it has also been suggested that bursts may result from the intrinsic evolutionary dynamics of viruses. Indeed, bursts could be caused by fitness valley crossing, or a neutral exploration of a fitness plateau until an escape mutant is found. In order to investigate the importance of these intrinsic causes of evolutionary bursts, we used a simulation software package to perform massive evolution experiments of viral-like genomes. We tested two conditions: (i) after an external change and (ii) in a constant environment, with the latter condition guaranteeing the absence of an external triggering factor. As expected, an external change was almost systematically followed by an evolutionary burst. However, we also observed bursts in the constant environment as well, albeit much less frequently. We analyzed how many of these bursts are triggered by deleterious, quasi-neutral, or beneficial mutations and show that, while bursts can occasionally be triggered by valley crossing or traveling along neutral ridges, many of them were triggered by chromosomal rearrangements and, in particular, segmental duplications. Our results suggest that combinatorial differences between the different mutation types lead to punctuated evolutionary dynamics, with long periods of stasis occasionally interrupted by short periods of rapid evolution, akin to what is observed in virus evolution.

## Introduction

Except for nucleocytoplasmic large DNA viruses (NCLDVs), which blur the line between viruses and cellular life forms, most viruses, especially those with RNA or single-stranded DNA (ssDNA) genomes, have notably compact genomes. These genomes are densely packed with information, often featuring overlapping reading frames and multifunctional proteins as common traits (Belshaw et al. 2008). Regardless of whether they have DNA or RNA genomes, viruses are submitted to very high mutation rates due to fast replication coupled with a lack of proofreading activity of their replicases (Sanjuán et al. 2010). Given their parasitic lifestyle, they undergo strong selection by the host’s immune system and competition among genetic variants. The evolution of viral populations has been shown to have complex multiscale dynamics, occurring in unpredictable bursts of mutation fixation (Bedford et al. 2011). These dynamics, which can lead to major outbreaks, pose a threat to human health (e.g. Ebola, severe Acute Respiratory Syndrome Coronavirus 2 (SARS-CoV-2), monkeypox, Dengue, Zika, etc.). They also jeopardize the sustainability of the food supply (e.g. avian influenza, foot-and-mouth disease, tomato spotted wilt disease, tomato yellow leaf curl virus, etc.). Therefore, understanding the potential causes of these bursts is essential to predict, prevent, and manage them. However, there is no consensus on their origin, with numerous hypotheses having been proposed in the literature. These hypotheses can be classified into two distinct groups, depending on whether the bursts have an exogenous or endogenous origin.

Exogenous causes correspond to events that occur in the environment of the virus. In this view, the evolutionary bursts are triggered by changes that occur outside of the viral population, like changes in the genetic composition of the host populations (Turner and Elena 2000, Morley and Turner 2017), hosts’ immune response developed against previously common variants (Elde et al. 2012), population bottlenecks due to random transmission events of very few particles (Escarmís et al. 2006), or new niche colonization (Stapleford et al. 2016). Other types of exogenous events include the interaction with other viruses during multiple infections, such as the acquisition of new genetic material through heterologous recombination. This phenomenon has been observed empirically (Parra 2019) and has been theorized as an efficient pathway to generate new phenotypes (Wagner 2011, Crona and Calafell 2018).

In contrast, endogenous causes do not invoke such events. In this view, bursts find their origin within the viral population and have an intrinsic evolutionary cause that can be explained using the fitness landscape and valley crossing metaphors. In the fitness landscape metaphor, initially proposed by Wright (1932), the population is viewed as evolving on a three-dimensional map, where horizontal coordinates represent the combination of phenotypic traits and altitude represents fitness. On this map, the population evolves through mutations and fitness increases until a local optimum is reached. The population is then stuck at this optimum because all mutants are deleterious and hence filtered out by purifying selection, resulting in evolutionary stasis. If there are any evolutionary mechanisms allowing the population to eventually leave this local optimum and cross a fitness valley, it can fix new mutations, triggering an evolutionary burst. By definition, valley crossing theories are conceptualized on a stable fitness landscape (Wright 1932) in the absence of any exogenous event. Valley crossing has been a matter of debate in the evolutionary community for decades.

Two main mechanisms leading to valley crossing have been proposed in the literature. However, there is no consensus on which one is the most common (Østman and Adami 2014). The first mechanism is obviously to move downhill: one or more deleterious mutations, which are not immediately filtered out by selection, lead part of the population to the depth of the valley where multiple alternative evolutionary paths are now available, including some giving access to a new peak of higher fitness. The likelihood of such valley crossing depends on many parameters, including the population size, the mutation rate, and the depth of the fitness valley (Kessinger and Van Cleve 2018). Various mechanisms could ease the process by lowering the strength of selection, such as population structure (Wright 1932), hitchhiking of deleterious mutations (Hill 2020), phenotypic stochasticity (Van Egeren et al. 2018), or stochastic tunneling (Iwasa et al. 2004).

The second mechanism is the ridge line. Several authors have pointed out that the importance given to fitness valleys is a direct consequence of the three-dimensional representation of adaptive landscapes (Gavrilets 1997). Indeed, in a high-dimensional space, many neutral paths are likely to connect fitness “peaks” to each other. In this view, the population can spread and wander within a neutral plateau until a fitter genome is found (Wilke 2001). Quasi-neutral landscapes that allow cryptic deleterious mutations have also been proposed (Masel 2006) and a general view of this phenomenon, called epochal evolution, was proposed by Crutchfield (2003).

Both exogenous and endogenous events are mentioned in the literature although exogenous events are generally given greater prominence (Ispolatov et al. 2019). Indeed, exogenous causes are often witnessed and are well studied experimentally (Morley and Turner 2017). Comparatively, endogenous events are difficult to identify *in vivo* and, by definition, cannot be experimentally triggered. The rareness of these events generates a need for more experiments. For instance, one can experimentally mimic specific mutations and examine the resulting landscape (Willemsen et al. 2016). Alternatively, researchers can simulate viral populations and compare the results with biological data (Bedford et al. 2011) or advance new models of evolution (Manrubia 2012). Here, we adopt a mix of both these approaches. Since endogenous bursts are, by definition, stochastic events that cannot be triggered by an extrinsic change (environmental or ecological), we performed large-scale *in silico* experiments, i.e. numerous repetitions of long-lasted evolutionary simulations in a constant environment, in order to isolate the few populations that had undergone an endogenous evolutionary burst. To understand the source of these bursts, we analyzed and compared them with those induced by environmental changes.

In our pursuit to pinpoint the molecular source of endogenous bursts, we employed Aevol, a simulation platform that accurately mimics genome architecture and the diverse mutational events experienced by viral sequences (Batut et al. 2013, Liard et al. 2020). Indeed, viruses evolve through a variety of mechanisms, not limited to single-nucleotide substitutions. More complex events, like insertions and deletions (referred to as indels), and structural variations contribute to their evolutionary process (Vignuzzi and López 2019). This includes the generation of extremely short genomes that lack coding capacity and act as parasites, relying on the wild-type virus for replication and encapsidation (Pita et al. 2007, Bhange et al. 2021, Rao et al. 2021).

It has been shown that, in Aevol, a high mutation rate and a large population generate short and compact genomes (Knibbe et al. 2007, Luiselli et al. 2024). Thus, by setting population size to 4096 individuals and the overall mutation rate to 7 × 10^−4^ mutations per base pair per generation (corresponding to 1 × 10^−4^ mutations per base pair per mutation type), a rate that is relatively close to RNA virus mutation rates (Drake et al. 1998, Sanjuán et al. 2010)), we were able to obtain virus-like genomes and to characterize their evolutionary dynamics. We performed two sets of 900 simulations each, starting from pre-evolved (hence well-adapted) viral-like sequences. In the first set of simulations, we slightly modified the virus environment, triggering exogenous bursts. In the second set of simulations, we simulated the same viruses but in a strictly constant environment. As it could be expected, in the latter situation, most populations fixed very few mutations with virtually no fitness gains. However, a few lineages substantially improved their fitness, even in this constant environment. Moreover, under both conditions, fitness gains occurred during short evolutionary bursts. We identified the endogenous events that triggered these evolutionary bursts and analyzed the importance of bursts depending on the kind of triggering events. Our results show that duplication events are at the origin of the strongest evolutionary bursts, echoing empirical studies and questioning the limitations of studying evolution with models based solely on point mutations.

## Materials and methods

### The Aevol platform

Aevol is a forward-in-time evolutionary simulation platform designed to study the evolution of genome structures (Knibbe et al. 2007, Batut et al. 2013, Liard et al. 2020). It uses an explicit representation of genetic information [sequence of nucleotides formalism (Hindré et al. 2012)] in which most elements in the genome (coding and non-coding sequences, promoters, operons, etc.) are represented and evolve in number and position under the pressure of a wide variety of mutational operators, including substitutions, indels, and structural variants (Banse et al. 2023). These features enable Aevol to model a wide variety of genomic structures representing different types of organisms, simply by changing the model parameterization. In the following paragraphs, we will give insights into the platform that are relevant for this paper. The next section presents how we used the model to simulate the evolution of viral sequences (a detailed description of the model can be found on http://www.aevol.fr).

#### Genome structure

In Aevol, the genome is a binary circular double-stranded sequence containing scattered genes and intergenic noncoding sequences, enabling the experimental study of the evolution of the genetic structure (typically the size of the genome, its coding proportion, the number and position of the genes, etc.) under different conditions (mutation rates, population size, etc.).

Conceptually, this genomic structure is similar to NCLDVs (i.e. members of the Megavirales order and of the Asfarviridae, Iridoviridae, Phycodnaviridae, and Poxviridae families) and to the double-stranded replication intermediates of small ssDNA viruses that belong to taxonomic families as diverse as the plant Geminiviridae and Caulimoviridae, the animal Anelloviridae, Circoviridae and Parvoviridae, and the bacterial Microviridae.

#### Genotype-to-phenotype-to-fitness mapping

The genome sequence is decoded to compute the phenotype and fitness of the individuals. The decoding process follows four steps: transcription, translation, protein folding, and protein–protein interactions.

##### Transcription:

The genome is first read to search for promoters and terminators. In Aevol, promoters are 22-bp-long consensus sequences, while terminators are small sequences able to form hairpin structures. The sequence between the promoter and the terminator is transcribed into an mRNA, with a transcription rate depending on the number of differences between the promoter sequence and the consensus sequence.

##### Translation:

Once all mRNAs are transcribed, Aevol searches for translation initiation signals. These are small 6-bp-long consensus signals representing ribosome binding sites, followed, 4 bp downstream, by a Start codon. Once such signal is found, the corresponding open reading frame is translated into a protein sequence until a Stop codon is found on the same reading frame. Importantly, this process allows for multiple different coding structures, such as operons, overlapping genes, and nested mRNAs, that are often found in viral sequences.

##### Protein folding:

**Figure 1.**
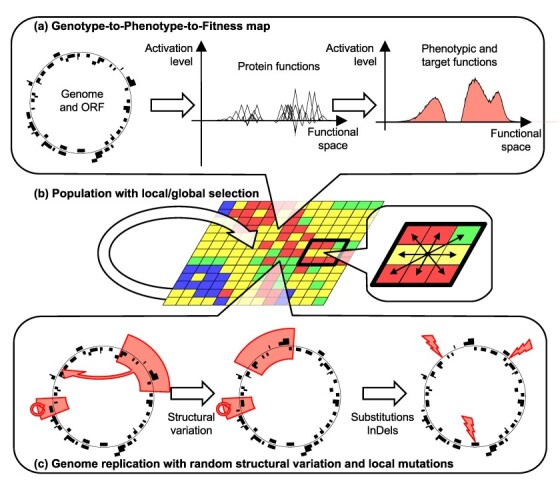
Overview of the Aevol simulation platform. (a) The genome codes for proteins. Proteins are represented as triangles in a mathematical functional space. The ideal phenotype (area filled in light red on the top-right subfigure) must be fitted as closely as possible by the combined effects of all proteins encoded in the genome. (b) The population is modeled as a grid of individuals. Reproductive competition occurs locally. (c) Mutations occur during reproduction. Mutations include both chromosomal rearrangements and local mutations (substitutions and indels).

**Figure 2.**
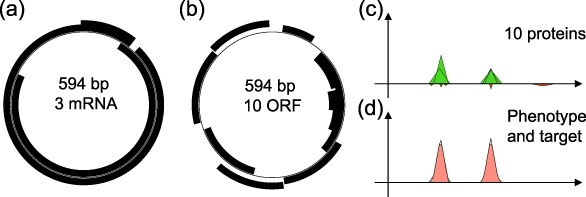
Example of a wild-type master sequence (Wild-type 2, corresponding to the simulation presented in Fig. 9). Left: Genome (thin circle) and the 3 mRNAs (black segments). Center: genome and ORFs (black segments). Top-right: activating (green positive triangles) and inhibiting (red negative triangles) proteins encoded by the 10 ORFs represented in the functional space of the model (see the main text). Bottom-right: phenotype (black line) and target function (red filled area).

When it comes to modeling the functional levels, Aevol switches to an abstract, mathematical representation of biological functions, hence enabling fast computation of phenotypes and fitness for a given genomic sequence. In this representation, biological functions are represented by numerical values $\phi $ in the [0,1] interval. The degree to which these functions are activated (or inhibited) can then be represented as $A\left( \phi \right) \in \left[ { - 1,1} \right]$, with $A\left( \phi \right) = + 1$ representing full activation and $A\left( \phi \right) = - 1$ full inhibition. In this formalism, a protein can be represented by three values, its main function $m$, the level of activation/inhibition of this function $h$, and its pleiotropic activity $w$ (the latter corresponding to functions close to the main one but less activated/inhibited, the activation/inhibition linearly decreasing with the distance down to 0 at distance $w$). Once the primary sequence of the protein has been obtained through translation, it is “folded” to compute the protein’s function, i.e. the three values $m$, $w$, and $h$. To this aim, each codon is assigned a binary digit corresponding to one of the three values. The “folding” of the primary sequence extracts the digit sequences corresponding to each parameter, enabling the computation of its numerical value (see http://www.aevol.fr for a detailed description of the folding process). Importantly, this process allows us to determine the functionality of a protein sequence whatever its length and composition. As a consequence, in Aevol, genes can have different sizes and mutations can affect both the composition and length of a gene, including by shifting the reading frame (e.g. by inserting or removing one or two nucleotides in a gene). After the folding process produced the three parameters, proteins can be represented by triangular-shaped functions in a 2D space where the first axis represents the function $\phi $ and the second axis represents the activation level $A\left( \phi \right)$ (Figs 1a and 2c).

##### Phenotype and fitness computation:

The whole proteome forms a network, with proteins sharing parts of their functions and interacting with one another. For the sake of computational efficiency, this is modeled as a linear interaction: all protein functions (activating or inhibiting) are summed up, with the resulting $\left[ {0,1\left] \to \right[0,1} \right]$ function representing the phenotype of the organism (Figs 1a and 2d). This phenotype is then compared to a reference function representing the ideal set of functions an organism can perform in its environment. This reference function is generally defined as the weighted sum of an arbitrary number of Gaussian functions and can be parameterized to represent more or less demanding environments (Liard et al. 2020). The reference function can also change at specific time points to simulate exogenous events. Finally, the fitness of a given organism is computed as a function of the difference between its phenotype and the reference function; the lower the difference, the greater the fitness. The intensity of selection is tuned by a parameter $k$; the higher $k$, the stronger the selection.

The decoding algorithm enables the computation of the fitness of an organism from its sequence. Importantly, this computation induces epistatic effects at different levels, including the genomic level (as genes can overlap on the genome—see e.g. Fig. 2b), the genetic level (due to codons interaction when folding the proteins) and the functional level since proteins influence several functions, possibly having beneficial effects on some of them and deleterious effects on others. Moreover, for a given protein, the beneficial/deleterious balance depends on the genetic context, i.e. on the presence/absence of other proteins contributing to the same functions.

#### Population structure and selection

Aevol uses a spatialized Wright–Fisher reproduction model with selection and mutation (Fig. 1b). Individuals are distributed over a square grid, and at each generation, they compete according to their fitness values to populate neighboring grid cells at the next generation. As with most models, population size is limited by computational load. The typical population size in Aevol is 1024 individuals distributed over a $32 \times 32$ grid. Note that, in accordance with the Wright–Fisher model, Aevol does not consider any mutation as lethal. However, organisms bearing mutations inducing large phenotypic variations (e.g. loss of important genes) usually have very low fitness and are virtually sterile.

#### Mutations

During replication, organisms may undergo different kinds of mutations. There are seven different kinds of mutations represented in Aevol (Fig. 1c). “Substitutions,” or point mutations, change a nucleotide into another. Given the binary sequence, in Aevol, substitutions change a 0 into a 1 or the other way around. “Small insertions” are mutations that add a short random sequence to the genome. In all the experiments presented here, the inserted sequence has a size of up to six nucleotides. “Small deletions” consist in the deletion of a small sequence of up to six nucleotides. “Duplications” are genome rearrangements that copy-and-paste a part of the genome. The copied sequence and the insertion locus are chosen randomly. “Deletions” are genome rearrangements that delete a random sequence from the genome. “Translocations” are genome rearrangements that consist in the selection of a random sequence which is extracted to form a circular plasmid that is in turn opened at a random position and inserted back into the genome at a random locus. “Inversions”: a random sequence is inverted, such that the first nucleotide of a strand becomes the last nucleotide of the complementary strand.

The mutation rate is defined as a probability for each nucleotide to initiate a mutation at each generation (usually between 10^−4^ and 10^−6^ mutations per nucleotide per generation for each type of mutation). This means that the mean number of mutations undergone by an individual directly correlates with its genome size. As a consequence, the genome size and structure are strongly influenced by mutation rates (Knibbe et al. 2007, Batut et al. 2013, Luiselli et al. 2024).

### Simulating viruses with aevol

As explained earlier, Aevol models generic organisms. However, depending on how the model is parameterized, it is possible to obtain organisms with particular genomic structures and genetic diversities in the population, which makes it possible to account for specific organisms Here we used a specific parameter set that enables us to model virus-like sequences. First, it has been shown that, in Aevol, high mutation rates lead to short, dense genomes with a high proportion of overlapping genes and very few noncoding sequences, akin to viral genomes with overlapping genes and multifunctional proteins (Knibbe et al. 2007, Batut et al. 2013). In all the experiments presented here, we used a mutation rate of 10^−4^ mutations per base pair and per generation for each of the seven types of mutations (resulting in an overall mutation rate of 7 × 10^−4^ mutations per base pair per generation). This results in a very high mutational pressure at the nucleotide level since several mutation types possibly affect several nucleotides at once (typically indels and chromosomal rearrangements). Second, at the functional level, viruses are characterized by a limited set of functions compared to cellular-based life. To account for this property, we used a simple reference function made of two independent Gaussians representing the structural and nonstructural genes involved in encapsidation/transmission and replication activities, respectively (Fig. 2d). This limits the epistatic interactions between these two functions (note, however, that epistatic interactions may exist at the genomic level, e.g. if genes encoding the two functions overlap). This property was also leveraged to simulate adaptation to a new environment by modifying the structural function (e.g. simulating the interaction of virions with novel and alternative cellular receptors) while keeping the replication function constant (see the “Experimental setup” section). Finally, since it has also been shown that, in Aevol, large population sizes and strong selection lead to compact genomes with less noncoding sequences akin to viral genomes, we used a large population size (4096 individuals on a $64 \times 64$ grid) and the strongest possible selection strength ($k = 2000$). These two parameters limit drift by lowering the quasi-neutrality threshold and increasing the selection coefficient of the mutations, so that fewer mutations fall below this threshold.

Using these parameters, the model spontaneously converges toward virus-like populations characterized by short yet information-dense genomes, limited biological functionality (at both the genetic and the phenotypic levels), and a large number of variant genomes akin to quasi-species together with strong selection acting on the master sequences (Frost et al. 2001, García-Arenal et al. 2003, Belshaw et al. 2008).

### Lineage tracking and analysis

#### Lineage tracking

During a simulation, Aevol tracks the characteristics of the best individual in the population for each generation. However, due to various classical processes in evolution (drift, clonal interference, selection for robustness, etc.), the lineage of the best organism at a given generation may eventually go extinct. To track the mutational history of the populations, Aevol keeps a perfect record of all replications (including the mutations that occurred during these replications). In an asexual population without recombination, this makes it possible to follow the exact line of descent of the final population, reconstruct all the genomes, and extract all the mutations on this line of descent. To ensure that we only consider fixed genomes and mutations (i.e. those descending directly from the coalescent), we systematically get rid of the last 5000 generations of the lineage (see the “Experimental setup” section).

As a result of the lineage tracking process, all the mutations, fitness values, and genome structures shown in this paper relate to individuals along the lineage of the final population. Importantly, at a given generation, this individual may not be the best individual in the population. But, as ancestors of the final population, they correspond to the true evolutionary history that led to the final population. For example, if a deleterious mutation occurs, it is very likely to be purged by purifying selection, hence being invisible in the lineage. However, if a deleterious mutation is present in the lineage (meaning it has not been purged by selection), it is very likely to have been reversed or compensated later on in the same lineage or to have hitchhiked with beneficial mutations (see e.g. Fig. 9 and related explanations in the main text). Tracking such forward dependencies enables unravel epistatic relationships through lineage analysis.

#### Computation of evolvability and mutation analysis

Using lineage data, we reconstructed all the genomes along the line of descent of the final populations and estimated their fitness and evolvability. In a broad sense, evolvability is the ability of an individual to adapt and evolve in the long run (while fitness corresponds to instantaneous adaptation). Here, we used an operational definition inspired by Woods et al. (2011). We measured evolvability as the expected degree to which a given genotype is likely to increase in fitness after a replication: for each genotype along the line of descent, we generated 10,000,000 independent offspring and evaluated their fitness in the same environment. We then computed evolvability as the fraction of beneficial offspring multiplied by their mean fitness improvement.

Since, along the line of descent, two successive genomes differ by a single mutation, we used the fitness and evolvability of the genomes to compute the mean variations of fitness and evolvability induced by each mutation type. Finally, for each type of mutation, we also computed the mean waiting times before and after a mutation of this type is fixed.

### Fitting mutational trajectories

To characterize the shape of evolutionary trajectories and classify them as stasis, gradual, or punctuated, we fitted them with mathematical models, using a methodology inspired by Wiser et al. (2013, 2018). We used three different models: Constant (${f_{\mathrm{C}}}$), Hyperbola (${f_{\mathrm{H}}}$), and Power law (${f_{\mathrm{P}}}$), representing stasis, bounded, and open-ended fitness increase, respectively (Wiser et al. 2013, 2018).

Formally, the three mathematical functions are


(1) $$\begin{array}{*{20}{c}}
{{f_{\mathrm{C}}}\left( t \right) = {f_{{\mathrm{init}}}}}\\
{{f_{\mathrm{H}}}\left( t \right) = {f_{{\mathrm{init}}}} + \frac{{a \times s\left( {t,{t_{{\mathrm{start}}}}} \right)}}{{s\left( {t,{t_{{\mathrm{start}}}}} \right) + b}}}\\
{{f_{\mathrm{P}}}\left( t \right) = {f_{{\mathrm{init}}}} + {{\left( {{b^{ - 1}}s\left( {t,{t_{{\mathrm{start}}}}} \right) + 1} \right)}^a} - 1}
\end{array},$$

where ${f_{{\mathrm{init}}}}$ is the fitness of the ancestor of the lineage and $s\left( {t,{t_{{\mathrm{start}}}}} \right) = \max \left( {0,t - {t_{{\mathrm{start}}}}} \right){\mathrm{\;}}$is a delay function that allows for a stasis period at the beginning of the lineages. The values of ${t_{{\mathrm{start}}}}$ allow us to distinguish gradual dynamics from bursty dynamics: gradual dynamics would lead to ${t_{{\mathrm{start}}}} \approx 0$, while, in a bursty evolution, the start parameter should be close to the first burst start.

As we suspect a saltational dynamics, we fit the mutational trajectories, meaning that we focus on the generations at which non-neutral mutations are fixed (more precisely, one generation before and one generation after each beneficial or deleterious mutation fixed in the lineage). This makes it possible to specifically adjust to the precise time points when trajectories switch from stasis to burst and to reduce the weight of long stasis periods as well as the weight of isolated mutations whatever their effect on fitness. Similar to Wiser et al. (2013), we fit these trajectories with the least square method using the lmfit Python package and use model selection to select the best of the three models. Since ${f_{\mathrm{C}}}()$ has no free parameter (${f_{{\mathrm{init}}}}$ being equal to the fitness of the ancestor) while ${f_{\mathrm{H}}}()$ and ${f_{\mathrm{P}}}()$ have three free parameters ($a$, $b$, and ${t_{{\mathrm{start}}}}$) and since ${f_{\mathrm{H}}}()$ and ${f_{\mathrm{P}}}()$ are non-nested models, we use a model selection based on the Bayesian Information Criterion (BIC), the preferred model being that with the lowest BIC value (Raftery 1995). This method allows us to select between different models without using *ad hoc* thresholds. It compares the different models with best fitted parameters and estimates which model is most likely to explain the mutational trajectories, penalizing the number of parameters of the models to take into account the fact that models with more parameters can always be better fitted to the data.

In the case of ${f_{\mathrm{H}}}()$ and ${f_{\mathrm{P}}}()$, the initial values of the parameters were set as follows: $b$, being homogeneous to a time, has been set to 1000. Given $b$, the $a$ parameter is initialized such that the curve reaches the maximum fitness value reached by the lineage at $t = 25,000$ generations (the end of the simulation). Finally, ${t_{{\mathrm{start}}}}$ was initialized to the generation of the first mutation fixed in the lineage.

To test whether punctuated evolution could fit our data, we tested a fourth model: a sum of hyperbolas ${f_{\mathop \sum H}}$. The rationale is that open-ended evolution could correspond not only to power law trajectories as proposed by Wiser et al. (2018) but also to successive hyperbolas. The former corresponds to open-ended evolution with diminishing return epistasis, and the latter corresponds to open-ended evolution with punctuated dynamics.

Formally, we define ${f_{\mathop \sum H}}\left( t \right)$ as


(2) $${f_{\sum H}}\left( t \right) = {f_{{\mathrm{init}}}} + \mathop \sum \limits_{i = 0}^{i < n} \left( {\frac{{{a_i} \times s\left( {t,{t_{{\mathrm{star}}{{\mathrm{t}}_{\mathrm{i}}}}}} \right)}}{{s\left( {t,{t_{{\mathrm{star}}{{\mathrm{t}}_i}}}} \right) + {b_i}}}} \right),$$

${f_{\mathop \sum H}}\left( t \right)$ has $3 \times n$ parameters. The choice of the best model is performed iteratively: for all simulations, we fit the mutational trajectory with one hyperbola ($n = 1$) and computed the BIC value. We then fit it with a sum of two hyperbolas ($n = 2$), if the model performs better with two hyperbolas than it does with one (i.e. the BIC value decreases), we continue with $n = 3$, and so on until the BIC value stops decreasing. This avoids the issue of multiple testing by only comparing one model to another. Note that because the trajectories are step-wise functions, they could be perfectly fitted with a sum of $M$ hyperbolas ($M$ being the number of fixed beneficial mutations). In order to avoid this caveat, we added a constraint to the parameters of the hyperbolas: the ${a_i}$ parameters should be at least a fifth of the total fitness increase. This constraint limits the total number of hyperbolas and allows for the identification of patterns of swift series of mutations (at each ${t_{{\mathrm{star}}{{\mathrm{t}}_i}}}$) followed by slow-down and stasis periods lasting up to the next hyperbola. In turn, it may cause a slight underestimation of the number of hyperbolic steps (as exemplified by the middle-left panel in Fig. 7).

### Identifying peak shifts and key innovations

In order to identify the precise events opening paths to a new fitness peak (i.e. peak shift triggering events), we need a formal definition of fitness peaks that allows the exact identification of the first mutant, which leaves the peak and whose offspring will eventually invade the whole population. Certainly, the idea of a mutational burst intuitively aligns with the concept of multiple advantageous mutations becoming fixed in a brief timeframe. However, this notion lacks precision for identification purposes, as it requires an analysis of mutation density and cannot be precisely associated with a specific triggering event.

We define a fitness peak (or a fitness plateau) as a point (or a set of points) in the fitness landscape where no genotype with a higher fitness is accessible through a single substitution (Wright 1932). It should be noted that this definition, implicitly used in most models because they generally consider only substitutions (Greenbury et al. 2022), neglects all evolutionary paths involving other types of mutations (in Aevol: indels and chromosomal rearrangements).

For each genome along the line of descent of the final population, we performed all possible point mutations and computed the fitness of the corresponding mutants. If none of these mutations resulted in a fitness improvement, the focal genome is considered to be on a “local peak” of the fitness landscape (peak here being understood in a broad sense, meaning that a fitness plateau is a peak with neutral mutations available). If at least one of these mutants has an increased fitness, the genome is not on a peak, which means that, if it has ever been on a fitness peak earlier in its evolution, it is likely to be shifting to a new peak. However, it is always possible that the genome is simply drifting transiently below the same peak. To filter out this situation, we extract the entire sequence of mutants between two peaks and consider only the peak shifts for which the fitness of the arrival peak is greater than that of the departure peak.

Following Erwin (2017) and Hochberg et al. (2017), we call a “key innovation” a mutation that leaves a fitness peak and initiates a sequence of mutations leading to another, higher, peak. Hence, key innovations are mutations for which the ancestor is on a peak (i.e. for which no favorable point mutation is available) and the mutant has access to at least one point mutation that would lead to a fitness higher than the original peak. In short, a key innovation is an endogenous event that triggers a peak shift.

Using this method, we identified all the peak shifts in our simulations and isolated the corresponding key innovations. We then classified the peak shifts according to the characteristics of the key innovations, deleterious mutations corresponding to fitness valley crossing, and neutral or quasi-neutral mutations corresponding to travelling along neutral ridges.

It is important to note that our method does not rely on any arbitrary threshold and is not dependent on mutation density or the fitness difference between two peaks. Hence, some peak shifts can be very short and contain few mutations, thus hardly corresponding to mutational bursts. To relate peak shifts and mutational bursts, we computed the number of favorable mutations and the fitness difference between the pre- and postpeaks. We then observed which types of key innovations are most likely to trigger large peak shifts corresponding to mutational bursts.

### Experimental setup

#### Wild-type evolution

We first used Aevol to evolve 30 populations of 4096 individuals with a mutation rate of 10^−4^ mutations per basepair per generation for each type of mutation and a target function containing the two viral functions at positions ${x_1} = 0.33$ (structural genes involved in encapsidation and transmission) and ${x_2} = 0.66$ (nonstructural genes involved in replication) in the functional domain of the model (see the “Simulating viruses in Aevol” section and Fig. 2). The 30 populations evolved for 200,000 generations in a stable environment in order to obtain 30 independent “wild-type” populations well adapted to their environment.

#### Evolution in a constant environment

We duplicated the wild-type populations to initiate 30 replicates per wild-type. The resulting 900 replicates evolved under the same conditions for 30,000 generations. We then reconstructed the lineages of the 900 replicates, suppressing the last 5000 generations in order to get rid of non-fixed events (see the “Lineage tracking and analysis” section). All the genomes, fixed mutational events, and peak shifts along the 25,000 generations of the lineages were then analyzed to search for endogenous mutational bursts and key innovations.

#### Evolution after an environmental shift

The same 30 wild-type populations were duplicated 30 times each, and the resulting 900 populations evolved in a new environment. To this end, we slightly shifted the position of the structural genes function from ${x_1} = 0.33$ to $x_1^{^{\prime}} = 0.3285$, keeping the replication function unaltered. This environmental change has been calibrated to induce a clear but limited drop in fitness (median fitness loss: 4.1 × 10^−2^). These 900 populations evolved for 30,000 generations, and the 25,000 first generations of the lineage were analyzed. Since these populations are not fully adapted to the new environment, they are expected to undergo exogenous evolutionary bursts starting at generation ${t_{{\mathrm{start}}}} \approx 0$.

## Results

### Wild-type populations

Given the very high mutation rates (see the “Experimental setup” section), wild-type populations are composed of a master sequence and a large cloud of mutants. After 200,000 generations of evolution, the master sequences are well adapted to their environment: the fitness values of the 30 master sequences range from 2.4 × 10^−2^ to 1.2 × 10^−1^ with a mean fitness of 5.6 × 10^−2^.

As expected under such a mutation rate, the genomes of the master sequences are short (median: 558.5 bp, range: 405–760 bp) with information-dense sequences, similar to viral sequences (Belshaw et al. 2007, Knibbe et al. 2007). On average, the master sequences contained 11.13 genes with an average of 2.9 noncoding base pairs per genome. Interestingly, most mRNAs are polycistronic (mean number of gene per coding mRNA: 3.5). Figure 2 shows an example of a wild-type genome and its phenotype.

### Fitness gain in the replicates

**Figure 3.**
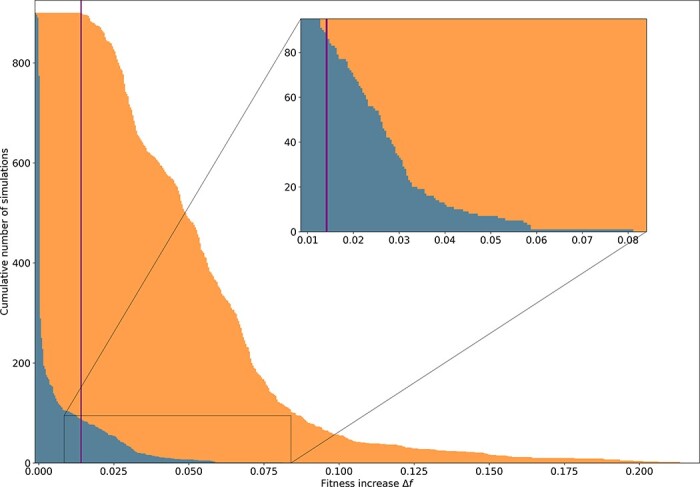
Cumulative decreasing histogram of the fitness increases for the 900 simulations in a constant environment (blue) and the 900 simulations in a new environment (orange). The sharp blue peak around zero corresponds to the majority of the simulations in a constant environment, showing no fitness gain ($\Delta f$ = 0) or drifting around their initial fitness value through quasi-neutral mutations. On the other hand, after an environmental change, all the simulations show a clear fitness gain with a minimal fitness gain after an environmental change of 1.4 × 10^−2^ (purple vertical line). Interestingly, 87 simulations in the constant environment (in blue) have a fitness gain greater than this limit, showing that some populations evolving under constant conditions can escape from their initial local optimum.

Starting from the wild-type populations, we ran 900 simulations in a constant environment and 900 simulations after an environmental change (see the “Experimental setup” section). Figure 3 shows the cumulative histogram of fitness gain after the 25,000 generations of the experiment for the two sets of simulations (blue corresponding to the fitness gain in a constant environment and orange to the fitness gain after an environmental change). All the simulations evolving in the new environment recover from the initial fitness loss, at least partly (median fitness gain: 5.3 × 10^−2^; Max: 2.1 × 10^−1^; Min: 1.4 × 10^−2^). Comparatively, the median fitness gain for the 900 simulations evolving in a constant environment is 2.1 × 10^−5^, most simulations showing no fitness gain, as illustrated by the sharp blue peak at zero in Fig. 3. This is consistent with wild-type populations being already well adapted to their environment and with the idea that environmental change is an exogenous triggering factor for evolutionary bursts. However, in constant environments, the distribution of the fitness gains also shows several fitness improvements of the same order of magnitude as those of populations adapting to a new environment (maximum fitness gain in a constant environment: 8.1 × 10^−2^, see the inset in Fig. 3). Indeed, among the 900 populations evolving in a constant environment, 87 show greater fitness gains than that of the worst population adapting to the new environment (purple vertical line in Fig. 3). This shows that some populations evolving under constant conditions can escape from their initial local optimum.

### Evolutionary dynamics of the replicates

**Figure 4.**
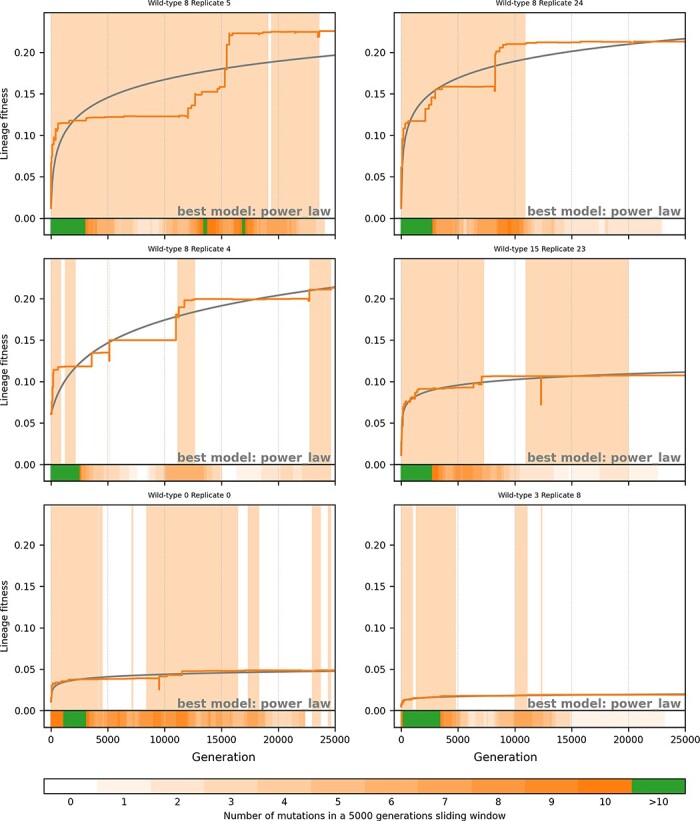
Six examples of lineages from simulations after an environmental change. Orange line: fitness along the lineage from generation zero to generation 25,000. Gray line: the best model using the methodology described in the Fitting mutational trajectories section. Shaded orange areas correspond to peak shift periods. Bottom of each graph: density of fixed beneficial mutations in a 5000 generations sliding window (see the legend). The six examples are (from top left to bottom right) the best simulation (maximum fitness gain between generations zero and 25,000), the 4th best, the 16th best, the 64th best, a random simulation (Wild-type 0, Replicate 0), and the worst one.

**Figure 5.**
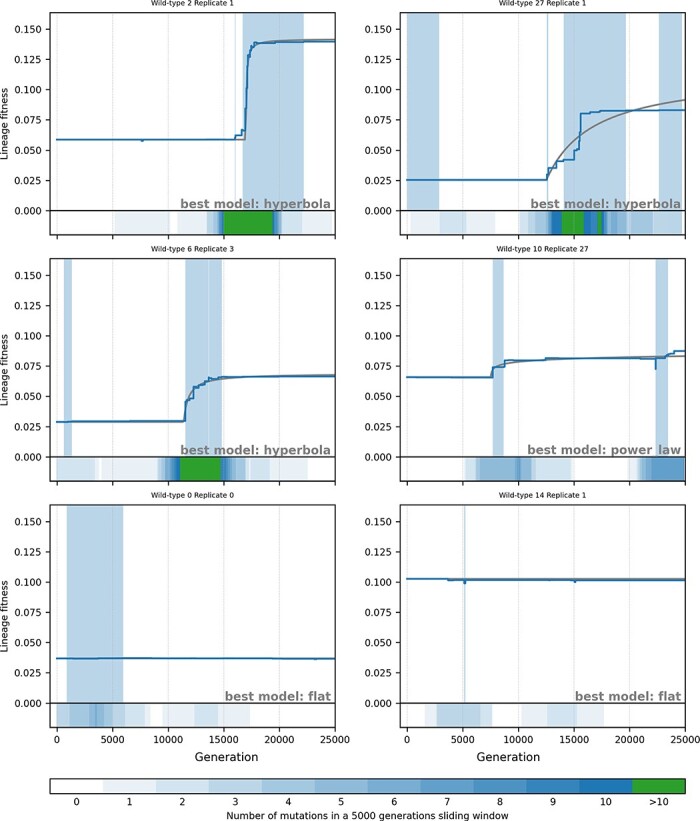
Six examples of lineages from simulations in a constant environment. Blue line: fitness along the lineage from generation 0 to generation 25,000. Gray line: the best model using the methodology described in the Fitting mutational trajectories section. Shaded blue areas correspond to peak shift periods. Bottom of each graph: density of fixed beneficial mutations in a 5000-generation sliding window (see legend). The six examples are (from top left to bottom right) the best simulation (maximum fitness gain between generations 0 and 25,000), the 4th best, the 16th best, the 64th best, a random simulation (Wild-type 0, Replicate 0), and the worst one.

Figures 4 and 5 show six examples of lineage trajectories after a change in environment and in a constant environment, respectively. In order to specifically observe those trajectories that have experienced substantial fitness gains, we plotted the 1st, 4th, 16th, and 64th best in terms of fitness increase (from top left to bottom right). In a constant environment (Fig. 5), these four lineages all show fitness gains larger than the minimal fitness gain in populations adapting to the new environment (1.4 × 10^−2^, purple vertical line in Fig. 3). We also show an example of a random simulation (first repeat of the first wild-type) and the worst simulation in terms of fitness gain. For all trajectories, we also show the density of mutation fixation along a sliding window of 5000 generations.

As expected, all the populations adapting to a new environment show a sharp increase in fitness and a high rate of mutation fixation at the beginning of the experiment (Fig. 4). However, this initial exogenously triggered evolutionary burst quickly vanishes with mutation fixation rates going down to almost zero after a few thousand generations although the best populations often show secondary bursts. The situation is completely different in the populations evolving in a constant environment. As expected under such conditions, there are no initial evolutionary burst and most trajectories are flat as illustrated by the random and worst replicates (Fig. 5, bottom left and bottom right, respectively). Nonetheless, bursts are clearly visible in the best populations, as exemplified by the four populations on top of Fig. 5 that all experience sudden increases in fitness and a high rate of beneficial mutation fixation in a short period of time.

### Analysis of the evolutionary dynamics

In order to quantify the visual intuition given in Figs 4 and 5 without relying on *ad hoc* thresholds, we fit the mutational trajectories with a flat function, a hyperbola, and a power law and select the best model using BIC values (see the “Fitting mutational trajectories” section). As expected, among the 900 simulations following an environmental change, none is best modeled by a flat function: 592 simulations are best modeled with a hyperbola and 308 with a power law. On the opposite, in the case of a constant environment, the majority of simulations are best modeled by a flat function (505 simulations), with 248 being best modeled by a hyperbola and 147 by a power law. Hence, as expected, most simulations in a constant environment can be considered in evolutionary stasis.

**Figure 6.**
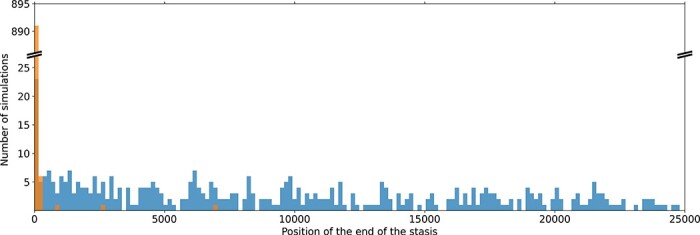
Histogram of the optimal ${t_{start}}$ values for the simulations best modeled with a hyperbola or a power law. In orange, the simulations with environmental variation: almost all the ${t_{{\mathrm{start}}}}$ values are close to generation 0 (99.4% are within the first 1% generations of the simulation). In blue, simulations in the constant environment: the ${t_{{\mathrm{start}}}}$ values are spread along all the 25,000 generations of the lineage. Note that the apparent slow-down of the number of optimal ${t_{{\mathrm{start}}}}$ in a constant environment (blue histogram) is essentially an artifact of the fitting process: in trajectories experiencing several bursts, only the first one is identified by the fitting process (see e.g. Fig. 5, middle-right panel).

Figure 6 shows the distribution of the ${t_{{\mathrm{start}}}}$ parameter for all hyperbolas and power laws after an environmental change (orange) and for a constant environment (blue). As expected, for almost all the simulations starting in a new environment, ${t_{{\mathrm{start}}}} \approx 0$, indicating that fitness starts increasing at the very beginning of the simulation. This is consistent with the theory: the environmental change lowers the fitness and reorganizes the fitness landscape, giving populations access to new paths for fitness improvement and hence triggering evolutionary bursts. However, more than half the trajectories (592/900) are best modeled by hyperbolas, showing that, in most cases, the exogenously triggered bursts quickly end and the populations enter a new stasis period.

This situation contrasts with the observed distribution of ${t_{{\mathrm{start}}}}$ for simulations evolving in a constant environment (excluding the 505 simulations best modeled by a flat function and for which ${t_{{\mathrm{start}}}}$ is not defined). In that case, the fitness increase seems to starts at random times during the simulation. This suggests that populations evolving in a constant environment were experiencing evolutionary stasis at the beginning of the experiment but that an endogenous event triggered a change in dynamics, further illustrating the punctuated nature of evolution in these populations.

### Identifying peak shifts

Previous results clearly show that the evolutionary dynamics is dominated by alternations of long stasis periods and rare evolutionary bursts triggered either by exogenous or endogenous events. So far, however, our results are either empirical, based on observation of the mutational trajectories and mutation fixation rates (Figs 4 and 5) or indirect, based on the distribution of ${t_{{\mathrm{start}}}}$ values in the mutational trajectory fits (Fig. 6). In order to understand what type of event triggers evolutionary bursts in our simulations, we need to be more precise and to pinpoint a specific mutation. For all the different genomes encountered along the lineages, we tested all point mutations and measured their fitness effect. This allows us to precisely identify peak shifts as periods of time during which at least one favorable substitution is immediately accessible from the tested genome. We remind that here, we define a fitness peak as a region of the fitness landscape where no favorable substitution is accessible regardless of any other type of mutation (e.g. chromosomal rearrangements; see the “Identifying peak shifts and key innovations” section).

Using this approach, we identified a total of 3631 peak shifts. Figures 4 and 5 show examples of peak shift periods for six simulations under the two tested conditions (shaded areas). They show that although our method often detects “shallow peak shifts” (i.e. peak shifts that do not correspond to substantial fitness gains), it captures most evolutionary bursts, whether they are defined by the density of mutation fixation or by fitness gains. As expected, simulations that started with an environmental change experience more peak shifts (mean: 3.16) than simulations in a constant environment (mean: 0.87, more than half the simulations having no peak shift at all). Even when removing peak shifts starting at generation zero (i.e. peak shifts triggered by a change in environment—that obviously bias the data—and peak shifts that started before the beginning of the experiment), the former experience significantly more peak shifts than the latter (2.19 vs. 0.70, Mann–Whitney *U* test, *P < *.05). This is consistent with our previous analyses of the mutational trajectories. Indeed, in a constant environment, most flat trajectories (373 over 505) had no peak shift at all. On the opposite, as might be expected, all simulations starting with a change in environment experienced at least one peak shift, with 873 being actually already shifting to a new peak at generation 0. This shows that environmental variation indeed triggers peak shifts in a large majority of populations. Note that in 27 experiments, the environment change does not trigger a peak shift. Although surprising at first sight, this can be explained simply: given the very high mutation rates, the initial populations contain numerous mutants, some of which possibly already adapted to the new environment. Here, one mutant in the population of wild-type 4 bears a mutation that is favorable in the new environment. This mutant is actually the ancestor of the 27 aforementioned lineages.

If we compare the characteristics of the peak shifts observed under the two conditions (again removing those starting at generation zero), we observe very similar dynamics. First, the duration of the peak shifts is very short compared to the total 25,000 generations of the experiments: after a change in environment, the median of peak shift duration is 1094 generations (InterQuartile Range, IQR: 2737) and it is 1260 generations in a constant environment (IQR: 3222). This shows that, under both conditions, populations spend more time on a peak than transiting to a new one. Similarly, there is little difference in the number of beneficial mutations fixed during a peak shift (both medians being equal to 2 mutations with IQRs of 3) or in the fitness gains between the pre- and postpeaks (medians: 1.5 × 10^−3^ and 0.5 × 10^−3^, IQRs: 5.0 × 10^−3^ and 3.6 × 10^−3^, respectively). Note that in all cases, distributions are highly skewed as shown by the difference between the medians and the means (mean duration: 2400 and 2798, respectively; mean number of mutations: 3.8 and 3.7, respectively; mean fitness gains: 5.4 × 10^−3^ and 4.3 × 10^−3^, respectively). This confirms the visual impression that most peak shifts are short and shallow and that only a subset of the peak shifts correspond to mutational bursts. Although all differences are significant (Mann–Whitney U test, *P < *.05), these values support the idea that peak shifts are similar in both kinds of experiments and suggest that the differences lie more in the frequency of the peak shifts than in their inner nature.

Indeed, the frequency of peak shifts can be roughly estimated by dividing the total duration of the experiment (900 × 25,000 generations in both experiments) by the total number of peak shifts (1971 and 626, respectively), showing that peak shifts are much more frequent after a change of environment than in a constant environment (one peak shift every 11,416 vs. 35,942 generations, respectively) as exemplified in Figs 4 and 5.

### Punctuated dynamics

Both our empirical observations of the lineages and formal characterization of peak shifts point toward punctuated dynamics. To further test this hypothesis, we modeled the mutational trajectories with more complex functions than the three previous ones, namely, sums of $n$ hyperbolas (note that sums of $n$ hyperbolas encompass both the constant function—when $n = 0$—and the hyperbola function—when $n = 1$—see the Materials and methods). Compared to the power law function that corresponds to open-ended dynamics with diminishing return epistasis (Wiser et al. 2013), a sum of $n$ hyperbolas would indeed correspond to punctuated open-ended dynamics.

**Figure 7.**
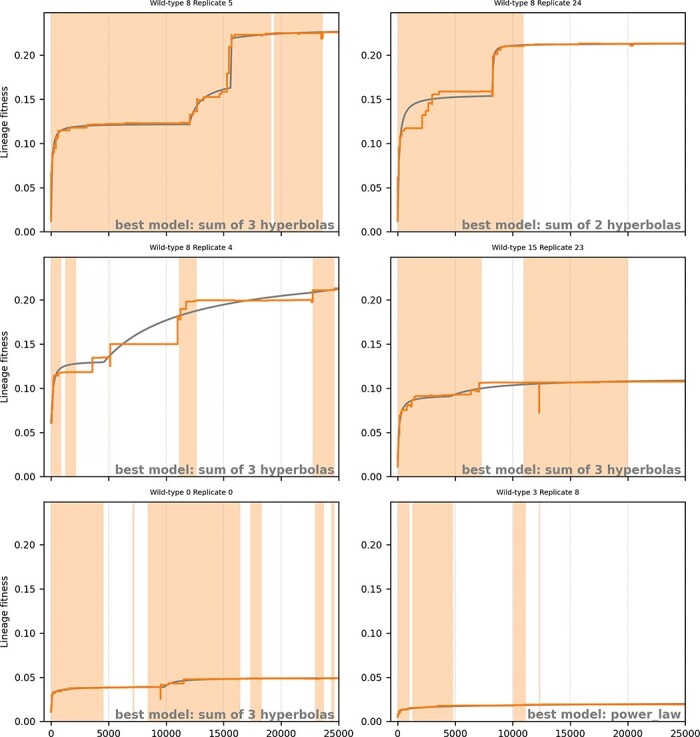
Sum of *n* hyperbolas models for the six lineages presented in Fig. 4 and corresponding to the best simulation, the 4th best, the 16th best, the 64th best, a random simulation (Wild-type 0, Replicate 0), and the worst one (all these simulations experienced an environment change at generation 0).

Figure 7 shows the same trajectories as Fig. 4 but with the sum of $n$ hyperbolas fits. It shows that most trajectories that were originally best modeled by power laws are actually best modeled by a sum of $n$ hyperbolas. Indeed, over the 1800 simulations, 504 are still best modeled by a flat function (zero hyperbola, all under constant conditions), but 1043 are now best modeled by a sum of $n$ hyperbolas ($n \ge 1$), while only 253 are still best modeled by a power law. This confirms that in our simulations, the evolutionary dynamics is mostly punctuated, with populations alternating between short evolutionary bursts and long periods of evolutionary stasis, with the former being triggered by either exogenous or endogenous events.

### Triggering events

Our formal characterization of peak shifts allows for the precise identification of the key innovations that triggered them (see the “Identifying peak shifts and key innovations” section), hence the nature of the peak shifts. Indeed, depending on the characteristics of the triggering events, one could distinguish valley crossing (triggered by a deleterious mutation) from traveling along neutral ridges (in which case the peak shift is triggered by a neutral or quasi-neutral mutation). To avoid direct and indirect effects of the initial environmental variation, we focused on the 900 populations that evolved in a constant environment. Among the 787 peak shifts observed in these experiments, we first excluded the 161 peak shifts starting at generation zero and for which the triggering event is not identifiable, since it has occurred in the lineage of a wildtype population. Indeed, as the initial populations contain clouds of mutants, it is possible that some of these mutants are already shifting at the beginning of the experiment. We also excluded the seven peak shifts starting with double mutations. Among the remaining 619 peak shifts, 383 have been triggered by a deleterious event and 61 by a neutral or quasi-neutral one (assuming a quasi-neutrality threshold of $1/N = 1/4096$). However, surprisingly, there are 175 peak shifts that were triggered neither by a deleterious nor by a neutral or quasi-neutral event but by beneficial events, more precisely by beneficial indels (105 events) and beneficial rearrangements (70 events)—beneficial substitutions being impossible, owing to the formal definition of peak shifts (see the “Identifying peak shifts and key innovations” section). These results show, first, that valley crossing is much more frequent than travelling along neutral ridges in our simulations. But, more surprisingly, they also show that a substantial fraction of peak shifts are triggered by beneficial events and that these events are complex mutations affecting more than one locus at a time.

**Figure 8.**
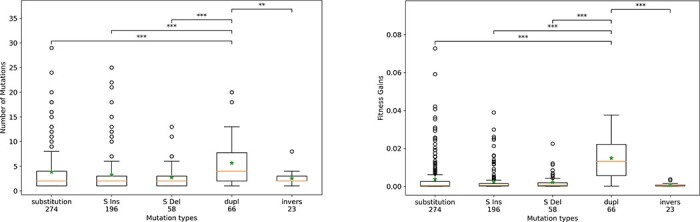
Left: Average number of favorable mutations fixed during a peak shift for the different types of key innovations (substitutions, small insertions, small deletions, duplications, and inversions) for the 619 peak shifts occurring in a constant environment. Right: Mean fitness gain of peak shifts for the different types of key innovations. Large deletions and translocations have been excluded because they do not trigger enough peak shifts (0 and 2, respectively). Mann–Whitney U-test with Bonferroni corrections for multiple tests, adjusted *P* < 2 × 10^−9^. All other paired tests were nonsignificant (adjusted *P* > .4).

As shown in Figs 4 and 5 and by the characteristics of the peak shifts (duration, number of mutations and fitness gain), not all peak shifts we observed correspond to mutational bursts. Indeed, peak shifts are highly variable in terms of size and intensity, from “shallow peak shifts” resulting in small fitness variations (see e.g. Fig. 5, wild-type 0, Replicate 0) to strong ones, resulting in large fitness gains, during which many mutations are fixed in a very short period of time (see e.g. Fig. 5, wild-type 2, Replicate 1). To further characterize key innovations, we computed, for each type of key innovation, the average fitness gain of the peak shifts it triggered as well as the average number of favorable mutations fixed during the peak shift. We first looked at the fitness gain depending on the sign of the mutation but found no major difference between deleterious, neutral, or beneficial key innovations: the average fitness gain of a peak shift is indeed 3.74(±0.8) × 10^−3^ when triggered by a deleterious mutation, 2.21(±1.7) × 10^−3^ when triggered by a neutral mutation, and 5.88(±1.2) × 10^−3^ when triggered by a beneficial mutation (± denotes 95% confidence intervals). However, when looking at the effect of the different types of mutation (Fig. 8), it immediately appears that, among all peak shifts, those triggered by segmental duplications result in fitness gains almost 10-fold larger than all the other ones and in the fixation of more beneficial mutations. These results show, first, that the “nature” of the peak shift (valley-crossing or neutral landscape) is less important than the type of mutation that triggered the shift and, second, that, among the different types of mutations, segmental duplications are by far the most likely to trigger strong peak shifts and mutational bursts.

### Analysis of the different types of mutation

Previous results have shown first that the evolutionary dynamics is punctuated, with most fitness gains concentrated in few peak shifts, and that duplications are more likely to trigger strong peak shifts than other types of mutations. To better understand the contribution of the different types of mutations to the evolutionary dynamics, we analyzed the 33,598 mutations that went to fixation in the 900 simulations in constant environments. Then, for each type of mutation, we quantified the number and fraction of events that triggered a peak shift (Nb.Trig.), the mean fitness effect of a mutation (∆Fitness), the mean time since the previous fixed mutation (∆*t*_pre_), the mean time before the next fixed mutation (∆*t*_post_), and the mean contribution to evolvability (∆Evolv.).

Table 1 summarizes the results for the seven different types of mutations. First, it shows the huge difference in the number of fixed events. Given that the mutation rate—hence the number of spontaneous mutations—is the same for all types of events (10^−4^ mutation per base pair per generation—see the “Experimental setup” section), this illustrates the difference in the distribution of fitness effects (DFEs) for the different types of mutations. Indeed, the number of substitutions (fixed point mutations) is three times greater than the number of fixed indels. On that matter, chromosomal rearrangements shows a striking pattern, with three types of rearrangements (duplications, deletions, and translocations) being hardly fixed at all, while the fixation rate of the fourth (inversions) is of the same order of magnitude as that of indels. This is due first to unbalanced rearrangements (duplications and deletions) being significantly more deleterious than balanced ones (translocations and inversions) and second to translocations being especially deleterious on dense genomes owing to their number of breakpoints. The situation is notably different for inversions, but one has to note that small inversions can have no effect whatsoever on the sequence (Trujillo et al. 2022), which increases their neutrality.

**Table 1.** Properties of the 33,598 mutations fixed in the 900 simulations in a constant environment.

Mutation type	Nb. fixed	Nb.Trig.	∆Fitness	∆*t*_pre_	∆*t*_post_	Confidence	∆Evolv.
Point mutation	17 387	274 (1.5%)	5.2 ± 3.2 × 10^−5^	642	646	–	−3.0 ± 10 × 10^−8^
Small insertion	4737	196 (4.1%)	1.0 ± 0.9 × 10^−4^	593	411	***	−4.0 ± 7.4 × 10^−7^
Small deletion	5117	58 (1.1%)	1.6 ± 0.7 × 10^−4^	404	590	***	−2.7 ± 1.5 × 10^−7^
Duplication	261	66 (25%)	3.4 ± 0.7 × 10^−3^	566	215	***	1.4 ± 1.4 × 10^−5^
Deletion	200	0 (0%)	9.1 ± 15.4 × 10^−5^	255	412	**	−7.4 ± 8.5 × 10^−8^
Translocation	130	2 (1.5%)	1.9 ± 2.4 × 10^−4^	773	632	–	−4.9 ± 10 × 10^−8^
Inversion	5766	23 (0.4%)	1.4 ± 0.9 × 10^−5^	914	900	–	2.0 ± 2.2 × 10^−8^

For each type of mutation, the columns show (from left to right) the number of occurrences in the 900 lineages, the number and fraction of mutations that triggered peak shifts (i.e. key innovations), the average effect on fitness, the average number of generations since the last fixed mutation, the average number of generations until the next fixed mutation, the significance of the difference, and the average contribution to evolvability (see the “Computation of evolvability and mutation analysis” section). ± values indicate 95% confidence intervals. *P < .05.** *P* < .01.*** *P* < .001; Mann–Whitney U test.

When looking at the fraction of fixed mutations that triggered a peak shift, it immediately appears that, although rarely fixed in the lineage, duplications are very likely to trigger a peak shift. Indeed, 25% of the fixed duplications are key innovations, a much higher fraction than for all other types of mutations. Moreover, ∆Fitness values show that fixed duplications are on average much more favorable than other types of mutations, further suggesting that, despite their very low fixation rate, duplications make decisive direct and indirection contributions to adaptation. Interestingly, while the 900 simulations starting with an environmental variation experienced more peak shifts than those evolving under constant conditions (2844 vs. 787), they also experience more duplications (722 vs. 261) and more peak shifts triggered by duplications (201 vs. 66), showing that duplications are also key events when adapting to new environmental conditions.

The two columns ∆*t*_pre_ and ∆*t*_post_, respectively, show the mean number of generations before and after a mutation of a given type. Overall, the mean waiting time between two mutations is 643 generations and any large deviation of ∆*t*_post_ from this value indicates whether a specific type of mutation, when fixed, changes the evolutionary dynamics. On the opposite, any large deviation of ∆*t*_pre_ indicates that the corresponding mutation type is preferentially fixed in a specific dynamic regime. Here, the effect of duplications is particularly pronounced with ∆*t*_post_ = 215 indicating a clear change of dynamics. Indeed, the fixation of a duplication triples the rate of fixation of mutations. This is to be considered with respect to the mean length of fixed duplications (64 bp—∼10% of the size of the genomes). This means that the strong increase in the rate of mutations fixation after a duplication cannot be explained by the direct effect of the (limited) increase in the size of the mutational target. Furthermore, the pattern is inverted for large deletions (∆*t*_pre_ = 255), indicating that large deletions are only likely to be fixed when occurring during an evolutionary burst. Also, note the very specific pattern of inversions, indicating that inversions are often fixed during long stasis periods, probably because, as explained earlier, they may have no effect at all on the sequence. Finally, when computing the mean effect of the mutation types on evolvability (column ∆Evolv. in Table 1), the effect of duplications appears even more clearly: duplications are the only type of mutational event that markedly increases evolvability. All other types of event either have a negligible contribution (substitutions, translocations, and inversions) or reduce evolvability (indels).

These results show that although their rate of fixation is extremely low (261 duplications fixed for a total of 900 experiments lasting 25,000 generations each), segmental duplications, when fixed, create new evolutionary opportunities and radically change the dynamics of evolution. It is tempting to invoke gene duplication to explain these observations (Ohno 1970). We thus analyzed the genetic content of the genomes before and after each of the 261 duplications fixed in our experiments. Results show that, out of these 261 events, only 11 were gene duplications and 126 resulted in the creation of a new functional gene (by duplicating segments of existing genes). However, among these 261 events, 201 resulted in modifications of an existing gene by adding new codons to an open-reading frame (ORF). Strikingly, duplications of sequences whose length is a multiple of three base pairs are over-represented (44 ± 6% vs. 29 ± 6% and 27 ± 5% for the two out-of-frame lengths), indicating a trend toward ORF lengthening without frame shifting.

### Illustration: Wild-type 2, Experiment 1

**Figure 9.**
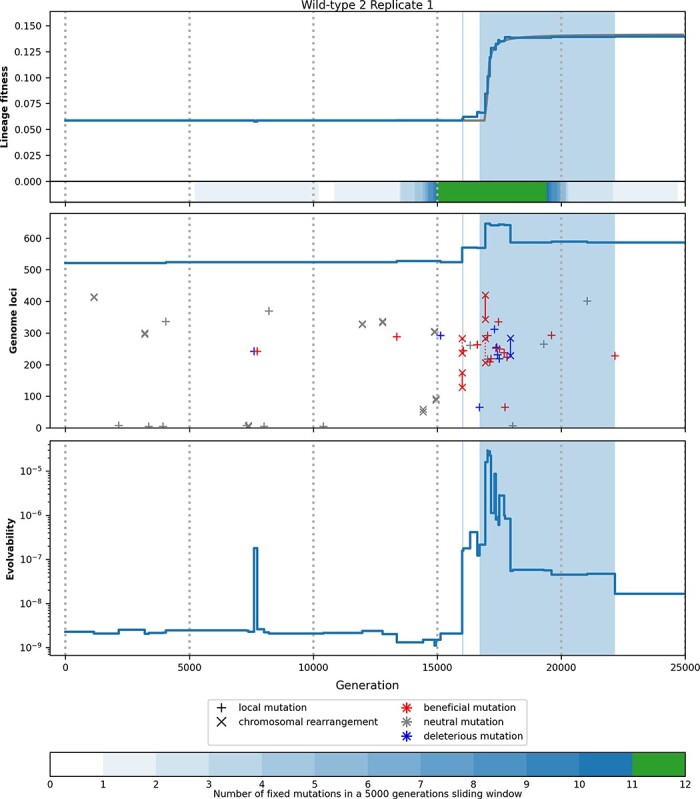
Example of key innovations in a constant environment. Top panel: Fitness of the lineage (as it is shown in Fig. 5) and density of mutation fixation. Middle panel: Mutations fixed in the lineage ordered by generations and loci (the blue line corresponding to the variation of genome size). Plus signs correspond to local mutations (point mutations and indels), segments ending with multiplication signs correspond to chromosomal rearrangements (duplications, large deletions, inversions, and translocations), and note that some segments are too short to be visible here. In the case of duplication, the origin segment is represented by a plain line and the inserted segment is represented by a dashed line. Blue symbols: deleterious mutations, red symbols: beneficial mutations, and gray symbols: neutral mutations. Bottom panel: Evolvability (see “Computation of evolvability and mutation analysis” section). Shaded areas correspond to peak shifts. These periods correspond to an increase in fitness, density of mutation fixation, and evolvability.

In order to illustrate the results presented earlier, Fig. 9 details the evolutionary dynamics of a specific experiment: Experiment 1 starting from Wild-type 2 and evolving in a constant environment (best fitness gain among the 900 simulations in a constant environment). The top panel shows the variation of fitness as well as the local density of mutation fixation (both being identical to the top-left panel in Fig. 5). The middle and bottom panels, respectively, show the mutations fixed during the experiment (identified by their types and loci, the line corresponding to the size of the genome) and the variations of evolvability during the 25,000 generations of the experiment. Shaded areas on the three panels show the two peak shift periods detected on this simulation.

As most simulations in a constant environment, this experiment starts by a stasis period. During this period, neutral mutations are occasionally fixed, most of them being short inversions (gray × symbols) or substitutions and indels (gray + symbols) at the rare neutral loci (especially at Loci 5 and 7). Notice the deleterious substitution at generation 7610 (blue + symbol) that is quickly reverted (red + symbol at generation 7731). The fixation of the deleterious mutation triggers a strong increase in evolvability since a favorable mutation is now immediately available. However, after the exact reversion of this mutation, evolvability drops back to its initial value. Note that, although meanwhile a favorable mutation is accessible, this episode is not considered as a peak shift because the deleterious mutation is exactly reverted.

The initial stasis period ends at generation 16,003 when the lineage enters a bursting period (as shown by the density of mutation fixation on the top panel). Shaded areas show the two peak shift periods. The first one is weak, stopping after 44 generations and only one favorable mutation. This first peak shift is triggered by a short beneficial duplication of a segment of 46 bp (plain red segment), inserted close to the original segment (dashed red segment). This duplication has a marginal effect on fitness, but it strongly increases the evolvability of the sequence (notice the log scale for evolvability) while initiating the peak shift. This first peak shift ends by the fixation of a beneficial substitution within the inserted sequence (red + symbol). A short pseudo-stasis period follows, during which two mutations are fixed within the inserted sequence: a neutral substitution (gray + symbol) and a favorable short deletion (red + symbol; notice the small variation in the genome size). Although this period is not detected as a peak shift (because no favorable substitution is directly available), it is characterized by a high evolvability value, showing that the evolutionary potential created by the first duplication is not yet exhausted. Indeed, a second peak shift immediately follows, which is more pronounced, starting at generation 16,702, lasting 5467 generations and containing 11 favorable mutations and 6 deleterious ones. The triggering event of the second peak shift is a deleterious substitution (blue + symbol) although the pattern is actually more complex. Indeed, this substitution has a marginal effect on fitness and on evolvability, and it is reverted at generation 17,729. Meanwhile, a second duplication follows the first one, inserting a new segment near the first inserted one and resulting in a second increase of evolvability. Together, both duplications increased evolvability by four orders of magnitude (from 2.1 × 10^−9^ to 2.2 × 10^−5^), creating a mutational hot-spot in the vicinity of the inserted segments in which several mutations went to fixation, either due to selection (favorable mutations) or to hitchhiking (deleterious mutations, including one large deletion that strongly reduced the evolutionary potential—plain blue segment). Finally, two favorable mutations (a small insertion and a substitution, both in the duplicated segment) end the sequence, further reducing evolvability and leading to a new period of stasis lasting 2831 generations, during which no mutations were fixed.

Although mostly illustrative, this example clearly shows how duplications interact with mutations of different types to generate punctuated dynamics. Indeed, scarce fixations of duplications add new genetic material to the sequence. On this new genetic material, further events of various types (including substitutions, indels and other rearrangements) can play, resulting in bursts of mutation fixation driven by selection and hitchhiking.

## Discussion

In this study, we investigated whether the mutation bursts that are observed in viral populations could be of endogenous origin or whether they are always triggered in response to exogenous events. To this end, we used the Aevol simulation platform to study the evolutionary dynamics of compact virus-like genomes in a constant environment or after an environmental change.

Our results show that, although environmental variations indeed almost systematically trigger bursts, punctuated dynamics could be observed even in the absence of such variations. Moreover, using a systematic exploration of the mutational neighborhood of the genomes along the line of descent, we were able to relate mutational bursts to peak shifts on the fitness landscape. Indeed, even though many of these peak shifts appear to be “shallow peak shifts” during which both the fitness gain and the number of fixed mutations remain low (therefore not corresponding to mutational bursts), a substantial fraction of the peakshifts we identified concentrate a large number of mutations, hence showing that peak shifting can result in mutational bursts. Having precisely identified peak shifts, we were also able to identify the type of mutational events that triggered them (the “key innovations”). Surprisingly, only a small fraction of these key innovations were neutral or quasi-neutral events, showing that, despite the very large number of dimensions of the search space, moving along neutral ridges is not frequent and that fitness valley crossing is the main peak shift mechanism. However, a striking result is that the largest peak shifts (those concentrating the greatest number of mutations and resulting in the biggest fitness gains) often begin with segmental duplications. This shows that these events, although rarely fixed in the lineage, are key events, likely to trigger mutational bursts. This is confirmed by an exhaustive analysis of all the mutational events that were fixed along lineages, as the fixation of a duplication appears to increase the rate of fixation of all types of mutations. Note that this increase in fixation rate could be thought to result from duplications increasing the size of the mutational target. However, this effect is not strong enough to account for the observed mutational bursts. We show that the main mechanism is an increase in evolvability caused by the duplications. This quickly leads to the fixation of several beneficial mutations and hence to the mutational burst.

Our results highlight the role of the complex interplay between different types of mutations. They show that even under conditions where the mutation rates are perfectly stable in the population, this interplay can result in a saltational dynamics in the rate of fixation of mutations. This dynamics is characterized by short periods of intense mutation fixation separated by long stasis periods. We propose that this dynamics is due to the difference in combinatorics of the various types of mutations. Combinatorics here refers to the number of different genomes that are accessible through a specific type of mutation. In other words, combinatorics quantifies the size of the mutational neighborhood of a genome for a given type of mutation. For instance, it is easy to show that, from an ancestral genome of length $L$, substitutions lead to a combinatorics of $3 \times L$ ($L$ possible loci times, three possible substitutions per locus). To the best of our knowledge, combinatorics is not considered as a key parameter in evolutionary biology. However, its importance becomes evident when we compare this simple formula with the size of the mutational neighborhood for more complex mutations. Indeed, consider, for instance, a segmental deletion in which a random subsequence of the genome is removed. The combinatorics of this mutation is now $L \times \left( {L - 1} \right)$: $L$ possible loci for the beginning of the deleted segment times, $L - 1$ possible loci for its end (for a circular genome). This shows that, for this kind of event, the size of the mutational neighborhood grows quadratically with the size of the genome, hence surpassing by orders of magnitude the size of the neighborhood of substitutions, even for small genomes like viral ones. Table 2 shows the combinatorics of the main types of mutations for a genome of size $L$ as well as the rationales of the computation.

**Table 2.** Combinatorics of several types of mutations.

Point mutations	$3L$	$L$ possible loci; 3 possible substitutions per locus
Small insertion (max length $l$)	$L \times \left( {\sum\nolimits_{1 < i \le l} {\left( {{4^i}} \right)} } \right)$	$L$ possible loci; ${4^i}$ possible random sequences of length $i$;$\sum\nolimits_{1 < i \le l} {\left( {{4^i}} \right)} $ possible random sequences of length lower or equal to $l$
Small deletion (max length $l$)	$L \times l$	$L$ possible loci; $l$ possible deletion lengths
Segmental duplication	$\left( {L \times \left( {L - 1} \right)} \right) \times L$	Two different breakpoints; one insertion point
Tandem duplication	$\left( {L \times \left( {L - 1} \right)} \right) \times 2$	Two different breakpoints; two possible insertion loci
Segmental deletion	$L \times \left( {L - 1} \right)$	Two different breakpoints
Translocation	$\left( {L \times \left( {L - 1} \right)} \right) \times L$	Two different breakpoints; one insertion point
Inversion	$L \times \left( {L - 1} \right)$	Two different breakpoints

The third column gives the rationales of the computation. Note that the formulas can change depending on specific mutational mechanics.

Given the large variations of combinatorics (from 3000 mutational neighbors for substitutions to 999,000,000 for segmental duplications, for a genome of length $L = 1000$ bp), it immediately follows that the time needed to explore a substantial fraction of the mutational neighborhood varies by orders of magnitudes depending on the mutation type. Hence, the overall evolutionary dynamics results from the juxtaposition of different mutational processes, each with its own timescale. Of course, the amount of exploration needed to find a beneficial mutation is also conditioned by how likely a given mutation type is to create a beneficial mutant, hence on the DFEs of the different types of mutations: mutation types with low combinatorics and most favorable DFE (typically substitutions) are the fastest, while mutations with high combinatorics and mildly favorable DFE (typically segmental duplications) are the slowest (mutations with unfavorable DFE—typically segmental deletions—being mainly fixed by hitchhiking). However, the combinatorics and DFE alone cannot explain punctuated dynamics, as they do not consider the interactions between the different types of mutations. Indeed, our results also show that some types of mutational events increase the probability of fixation of others through an increase in the overall evolvability. Hence, the punctuated dynamics observed in our simulations results from the interplay of two mechanisms: mutation combinatorics and the probability of beneficial mutants, which lead to very different time scales, and evolvability that reignites the fixation of fast events after the fixation of slow ones, meaning that the DFE of fast mutations depends on the occurrence of other mutations (typically duplications). Ultimately, this interplay leads to the fixation of few slow events and many fast ones: in our simulations, the rate of fixation of substitutions is more than 50 times higher than that of duplications, despite their same spontaneous mutation rates (Table 1). The occurrence of a slow event opens new favorable evolutionary pathways and mutations that have the most favorable combinatorics/DFE balance quickly accumulates as they allow fast exploration of these new pathways.

Crucially, this theoretical understanding of the behavior of the model is not dependant on the details of the genome structure. We conjecture that this dynamics of bursts and stasis can arise in any population provided that there are multiple mutation types, with different timescales and with different effects on the sequence. Indeed, on a rugged fitness landscape, fitnesses are locally correlated, but correlation vanishes at medium distances. Consequently, when a population is stuck on a local optimum (meaning the local correlations are all unfavorable), a duplication can partially reset correlations, creating new opportunities for local mutations. This phenomenon is particularly visible in our simulations, probably because the compact genome structure makes it possible for populations to quickly reach local optimums, hence clearly separating the different time scales. A key question is to determine whether other characteristics of our simulations also contribute to emphasizing this mechanism. In particular, it is widely recognized that the probability to cross fitness valleys is higher at a low effective population size because it allows for a high level of drift (Lande 1985). Here, the population size is rather small compared to real virus populations [although viruses effective population sizes could actually be much lower than census population sizes due to recurrent bottlenecks (Zwart and Elena 2015)], and one could legitimately wonder whether this could be at the origin of the observed saltational dynamics. However, in our simulations, the level of drift is actually limited by the high selection strength *k*, which widens the DFEs of the mutations, hence reducing the fraction of mutations falling below the drift barrier. Moreover, a substantial fraction of the mutational bursts we observe are triggered by favorable events, showing that drift is not the main factor explaining our observations. Even if this is arguably a challenging task (Conrad and Hurles 2007), our results call for the development of mathematical population genetic models including multiple types of mutations to assess whether they would predict a similar dynamics.

In our simulations, duplications are by far the most beneficial type of chromosomal rearrangement when it comes to triggering evolutionary bursts (Fig. 8 and Table 1). A natural hypothesis would be that these events lead to gene duplication divergence (Ohno 1970, Zhang 2003, Gao et al. 2017). However, an in-depth analysis of the duplication events shows that only a small fraction of fixed events corresponds to gene duplications. On the opposite, most fixed duplications actually add a small segment to extant genes. Table 1 suggests that the advantage of duplications comes both from their direct effect on fitness (on average, fixed duplications are more beneficial than any other type of mutation) and from their indirect effect on evolvability (as duplications are the sole type of mutation that substantially increases evolvability). This suggests that, in addition to combinatorics, the mechanistic properties of the different types of chromosomal rearrangements play a role in their fixation and in their burst-triggering probabilities. Classically, rearrangements are classified as “unbalanced” or “balanced” depending on whether they change the genome length (duplications and deletions) or not (translocations and inversions (Mérot et al. 2020)). Our results suggest that an important factor is whether rearrangements are conservative or not, with conservative events being those keeping the rearranged segment intact (typically duplications), while non-conservative ones modify it (typically deletions and translocations). We propose that the advantage of duplications in triggering evolutionary bursts comes from the fact that they are conservative and unbalanced. Being conservative limits how deleterious the events are (duplications being only harmful at their insertion point), while being unbalanced creates a strong potential for other types of mutations to modify the inserted sequence, hence the gain of evolvability and the subsequent burst of mutation fixation, including substitutions, indels and large segmental deletions that remove parts of the inserted sequence (Fig. 9 and Table 1). Hence, our results support the genomic accordion model (Elde et al. 2012, Filée 2013) but show that the accordion can play a role even in a constant environment and that it is not restricted to gene copy number variations. Indeed, although gene duplications are frequent and play a major role in double-stranded DNA viruses (Elde et al. 2012, Gao et al. 2017) and have been observed in long RNA viruses (e.g. Citrus tristeza virus (Kang et al. 2018)), they are rare in RNA viruses. However, it is worth noting that small-sized duplications, similar to those we observed in our simulations, have been shown to lead to significant increases in fitness and mutation rate in hepatitis C virus (Le Guillou-Guillemette et al. 2017), respiratory syncytial virus (Schobel et al. 2016), and more recently in SARS-CoV-2 (Zhou et al. 2023). Furthermore, in an exhaustive recent study, Aguilar Rangel et al. (2023) combined new cutting-edge sequencing technologies with bioinformatics analyses to characterize the nonpoint mutation component (i.e. duplications and indels) of the mutational spectra of polio and dengue viruses. They found that short indels (*<*10 nucleotides) represent a major, previously unseen, component of the mutational spectra. Some of these indels were actually positively selected; in general, these beneficial indels did not affect secondary RNA structures with regulatory functions nor resulted in out-of-frame mutations at the protein level, hence showing the same bias in favor of in-frame insertion lengths as observed in our results. Another very interesting observation by Aguilar Rangel et al. (2023) was that the rates of indel production in Dengue virus was host species dependent, with more indels fixed in the host mosquito than in the alternative host human. Such host-dependent differences in the abundance of indels has also been shown in other viruses [e.g. broad bean mottle virus (Llamas et al. 2004), tomato black ring virus (Hasiów-Jaroszewska et al. 2018), and, more recently, in two different betacoronaviruses (Hillung et al. 2024)], suggesting the participation of cellular factors in the replication of RNA viruses and genesis of defective genomes. Interestingly, we observe similar differences in the rate of fixation of duplications between the experiments in constant and changing environments, suggesting that, alternatively, these differences could be rooted in the degree of adaptation of the viruses to their host.

Viroids are subviral plant pathogens formed by small circular noncoding RNA molecules (between 250 and 400 nucleotides long) (Flores et al. 2014). They represent an excellent model to study the effect of accordion evolution in small RNA molecules. Interestingly, several of the known viroid species accumulate longer genomes generated by partial duplications during long-term infections, which closely resembles the dynamics observed in our simulations. These longer variants are, however, displaced by their short versions at the onset of new infections. For example, the coconut cadang-cadang viroid exists in two different forms, the so-called fast and slow, with the second containing a 41-nucleotide duplication (17% of the genome length). A more extreme duplication is the generation of Coleus blumei viroid 3 (361 nucleotides long) from Coleus blumei viroid 1 (only 248 nucleotides long). Sanjuán et al. (2006) showed that, in all known cases of enlarged viroid genomes, the long variants were more robust to mutations than the short ones, despite both having one-step neutral mutational neighborhoods of similar size. This observation suggests that it would be interesting to extend the study of our simulations to the measurement of robustness in addition to evolvability.

Given that in our simulations, 25% of fixed duplications trigger peak shifts, it would be tempting to conclude that segmental duplications predict mutational bursts. However, it is important to keep in mind that we analyzed fixed events, hence being dependent on survivorship bias: the fixation probability of a key innovation depends on the fitness gain of the following peak shift that cannot be predicted. This is especially true for segmental duplications, as they bear an indirect burden owing to the increase in genome size (Willemsen et al. 2016). This may explain why the fraction of fixed duplications triggering peak shifts is so high and why duplications trigger higher peak shifts: only those duplications that are followed by large mutational bursts come to fixation by hitchhiking the burst they, themselves, triggered.

In this study, we used the Aevol model which is based on a generic DNA-based representation of the genome structure and of the genotype-to-phenotype map. This model does not intend to represent specifically a particular organism. On the opposite, it lets evolution determine the structure of the genome depending on the main evolutionary parameters (mutation rates, population size, target function, etc.). This of course raises the question of whether our results can apply to specific viral species given the diversity of nucleic acid sequences present in viruses. First, by using specifically tuned parameters, the model has been able to evolve short and dense genomes, akin to viral ones. Hence, the encoding of genetic information is close to that observed in viruses and the gross effect of the different mutation types is likely to be close to that observed in viruses too. Second, we would like to argue that the dynamics we observe are more rooted in the diversity of the mutational operators than in the specifics of the nucleic acid on which information is encoded: as long as the nucleic acid is possibly altered by both local and segmental events, the saltational dynamics we observe could be at work. However, it is of course possible that, in a real viral population, other types of sequence alteration (e.g. segment reassortment or recombinations) play a role similar to that of duplications in our simulations. It is also possible that the saltational dynamics be less marked than in our simulation or hidden by other factors such as environmental variations. However, we think that our results provide important theoretical insights into the evolution of viruses under the joint pressure of several mutational operators.

On a more general level, our results open intriguing questions on the interpretation of the fitness landscape metaphor and on the related question of fitness valley crossing. Several authors have argued that the classical 2D representation of fitness landscapes is misleading and that in large dimensions, fitness landscapes are likely to be “holey landscapes” in which many neutral ridges or neutral landscapes connect fitness peaks (Gavrilets 1997, Wilke 2001, Crutchfield 2003, Fragata et al. 2019). Our results first show that very few key innovations are neutral or quasi-neutral. Although this can be due to the specifics of the Aevol genetic code in which there are no synonymous codons (hence less neutrality), this observation suggests that neutral landscapes are not an efficient way to escape local fitness peaks. Hence, our results support the findings of Chatterjee et al. (2014) that showed that the time needed to explore the neutral basin grows exponentially with the size of the genome, mechanistically limiting the evolutionary potential of neutral landscapes. Interestingly, Chatterjee et al. (2014) also suggest that chromosomal rearrangements would allow reducing the search process to a polynomial one in the size of the duplicated sequence.

Even more interestingly, our results suggest that the fitness landscape metaphor is biased not only by the classical 2D representation but also by its mere topological representation. Indeed, the “surface” of the landscape is composed of local mutations: substitutions, and sometimes indels. But the metaphor does not allow the representation of other kinds of mutational events and, in particular, chromosomal rearrangements, which would look like “jumps” over the landscape. Moreover, as shown by combinatorics, the number of possible jumps is much greater than that of local steps. Metaphorically speaking, every point in the landscape is an airport, weakly connected to its neighbors not only by a few roads (substitutions) but also by direct flights (rearrangements) to a very large number of other airports all over the world. Because most jumps are deleterious, the local connections are those that drive short-term behavior. However, we conjecture that in such a “multimutational fitness landscape,” the number of available jumps is such that there are virtually no fitness valleys that cannot be jumped over by a chromosomal rearrangement.

Interestingly, the dynamics we observe in our simulations are very similar to those conjectured by Kauffman and Levin (1987) when “correlated” mutations (with small fitness effects) and “uncorrelated” mutations (for which the fitness of mutants does not correlate with the fitness of their ancestors) coexist. According to Kauffman and Levin (1987), in such a case, evolution proceeds in three phases: initially, uncorrelated mutations have the best chance to increase fitness. Then, on the midterm, mutations that correlate allow for fine-tuning. Finally, in the long term, the population is stuck waiting for a beneficial uncorrelated mutation to make a “jump” through the fitness landscape. However, our simulations suggest that not all rearrangements are evenly “uncorrelated” and that duplications are the most likely to make favorable jumps.

In this study, we focused on very short, dense genomes and showed that punctuated evolution similar to that observed in viruses can emerge in a stable environment with very few assumptions about the evolutionary process. This raises the question of the evolutionary dynamics of longer, possibly less dense, sequences (e.g. prokaryotic genomes) under the mixed effect of fast and slow mutations. Indeed, alternations of burst and stasis are mainly due to the difference in tempo between fast fixation of beneficial local mutations and long waiting times for key innovations. In this view, increasing genome size will increase both the time needed to reach a peak and the time needed to explore the duplication neighborhood. However, combinatorics shows that both will not grow at the same rates (Table 2). A direct consequence is that, under similar evolutionary conditions (mutation rates, population size, etc.) the evolutionary dynamic is likely to be very different depending on genome size. Exploring the interplay of the different types of mutations for various genome structures constitutes a very promising perspective of our work. In particular, we conjecture that the punctuated dynamics that we observed in compact virus-like genomes will be less marked or even absent in longer sequences owing to the time needed to reach a marked stasis period (i.e. the time needed to explore all possible substitutions). Yet, even if the dynamics are different, the potential of rearrangements as key innovations is likely to still be present. Indeed, duplication-triggered evolutionary bursts have been observed in *Escherichia coli*, in which the partial duplication of a promoter triggered an evolutionary burst, resulting in novel functions (Blount et al. 2012). One could also question whether, in longer and less information dense genomes, other types of rearrangements could play the same role as duplications in our simulations. In particular, inversions, which have little to no effect in our simulations (Table 1), may play a more important role in other kinds of genomes owing to their limited deleterious effect coupled with a reasonably large combinatorics (Trujillo et al. 2022). Exploring these dynamics constitutes an immediate perspective of our work although our methodology is probably not suited to longer genomes, as they may never reach the top of a local peak due to much longer exploration times.

An intriguing question and exciting perspective of our work would be to link combinatorics of mutations with micro/macro evolution. Indeed, our results suggest that, depending on the timescale, evolution will not show the same kind of dynamics. The idea is that, on short time scales, microevolution would be limited to the context of optimization of a new trait or to a new environment, mainly via fast mutations (substitutions and indels), but that, on long time scales, macroevolution would be mainly driven by rare bursts of innovations triggered by slow mutations (chromosomal rearrangements) that would maintain the microevolution active on the long run despite diminishing return epistasis (Banse et al. 2023). This hypothesis, aimed at bridging the gap between long-term and short-term evolution, is reminiscent of the proposal of Uyeda et al. (2011) where a corpus of biological and archaeological data is fitted with different models of long-term evolution, suggesting multiple evolutionary bursts that the authors attribute to environmental changes. Our results suggest that these bursts could also have an endogenous origin, with slow mutations occasionally triggering innovations.

## Data Availability

Aevol is available freely at https://www.aevol.fr. All simulated data are available upon request from the corresponding author.
